# Impacts of Non-alcoholic Fatty Liver Disease on Acute Coronary Syndrome: Evidence and Controversies

**DOI:** 10.1007/s11883-023-01146-7

**Published:** 2023-09-28

**Authors:** Shun-Yi Shi, Fang Jia, Meng-Fei Wang, Ya-Feng Zhou, Jian-Jun Li

**Affiliations:** 1 Department of Cardiology, The Third Affiliated Hospital of Soochow University, Changzhou, China; 2 Department of Cardiology, Suzhou Dushu Lake Hospital, Dushu Lake Hospital Affiliated to Soochow University, Medical Center of Soochow University, Suzhou, China; 3 Cardio-Metabolism Center, Fu Wai Hospital, Chinese Academy of Medical Sciences, Peking Union Medical College, BeiLiShi Road 167, Beijing, 10037 China

**Keywords:** NAFLD, ACS, Mechanisms, Outcomes

## Abstract

**Purpose of Review:**

Acute coronary syndrome (ACS) and non-alcoholic fatty liver disease (NAFLD) are two clinically common disease entities that share numerous risk factors. This review aimed to discuss the impacts of NAFLD on ACS.

**Recent Findings:**

In an era of improved control of traditional risk factors, the substantial burden of cardiometabolic abnormalities has caused widespread concern. NAFLD is considered the hepatic component of metabolic syndrome, which can exert an impact on human health beyond the liver. Accumulating studies have demonstrated that NAFLD is closely related to cardiovascular disease, especially coronary artery disease. Interestingly, although recent data have suggested an association between NAFLD and the incidence and outcomes of ACS, the results are not consistent.

**Summary:**

In this review, we comprehensively summarized evidence and controversies regarding whether NAFLD is a contributor to either the development of ACS or worse outcomes in patients with ACS. The potential pathophysiological and molecular mechanisms involved in the impacts of NAFLD on ACS were also elucidated.

## Introduction

Acute coronary syndrome (ACS), a thrombotic, dramatic, and life-threatening complication of atherosclerosis, remains one of the leading causes of death [1], although substantial progress has been made in the diagnosis and treatment of this devastating disease [2, 3].

Over the past few decades, epidemiological shifts in ACS have occurred. On the one hand, there has been a decline in the incidence of ST-segment elevation myocardial infarction (STEMI) and an increase in non-ST-segment elevation myocardial infarction (NSTEMI), which might be partially explained by changes in plaque biology [4]. Plaque erosion with an intact fibrous cap now accounts for approximately one-third of ACS cases and up to two-thirds of NSTEMI cases due to the widespread intense control of lipid levels and other traditional risk factors [4, 5]. On the other hand, acute myocardial infarction (AMI) patients are increasingly younger, with a high prevalence of cardiometabolic comorbidities and a 1-year mortality rate approaching 10% [6]. Obesity, consequent insulin resistance, and adverse lifestyles are on the rise worldwide, contributing to an elevated prevalence of cardiometabolic disease [7].

Non-alcoholic fatty liver disease (NAFLD) is the hepatic manifestation of systemic metabolic syndrome (Mets). It is the most common cause of chronic liver disease globally, with an estimated prevalence of 25%, putting a significant health and economic burden on all societies [8, 9]. NAFLD represents a clinically heterogeneous disease entity, with a disease spectrum ranging from simple steatosis, non-alcoholic steatohepatitis (NASH), and non-alcoholic liver cirrhosis to hepatocellular carcinoma (HCC) [10]. It is a multiple-system disease with an impact that extends beyond the liver. Most patients with NAFLD are affected from the early stages of the disease and are often comorbid with other cardiometabolic risk factors [11]. Cardiovascular events are the most common cause of mortality in patients with NAFLD, although a causal relationship has not yet been established [12]. There are likely multiple underlying mechanisms by which NAFLD increases the risk of cardiovascular disease (CVD), including altered lipid metabolism, systemic inflammation, systemic insulin resistance, oxidative stress, endothelial dysfunction, and plaque formation/instability [13].

In addition, due to the close connection between NAFLD and metabolic syndrome, it has been suggested recently that this kind of disease should have a new name—metabolic dysfunction-associated fatty liver disease (MAFLD) [14, 15]. The diagnosis of MAFLD is based on the coexistence of hepatic steatosis and one of three additional criteria, namely overweight/obesity, type 2 diabetes mellitus (T2DM), or evidence of metabolic abnormalities [14, 15]. Studies have concluded that refining NAFLD as MAFLD may identify more individuals with an increased risk of CVD and improve clinical utility [16, 17].

For many years, NAFLD was thought to be benign and without significant clinical importance. However, increasing recognition of this disease entity and its strong relationship with CVD have aroused an interest in further investigation. To date, data on the role of NAFLD in ACS are limited and inconsistent. In this review, we focused on updated information regarding the impacts of NAFLD on ACS. We attempted to provide a different perspective on the plausible underlying mechanisms and shed some light on areas of further research.

## Methods

We performed a narrative review of the literature in the PubMed database. Study selection included cross-sectional, case–control, cohort, or retrospective studies. We searched titles or abstracts for the terms “acute coronary syndrome” OR “myocardial infarction” AND “non-alcoholic fatty liver disease” OR “non-alcoholic steatohepatitis” OR “metabolic dysfunction-associated fatty liver disease.” English-language publications were screened. There were no time restrictions for eligible studies.

## Impacts of NAFLD on ACS

The impacts of NAFLD on ACS are extremely complex and multidirectional. Studies of this issue have shown inconsistent results.

Currently, the gold standard for the diagnosis of NAFLD is liver biopsy, but it is not widely used due to its invasive nature, high cost, and inconvenience. In clinical practice, the commonly used methods for NAFLD identification are imaging modalities, including ultrasound and computed tomography (CT), as well as some non-invasive surrogates or biomarkers.

### NAFLD and Prediction of ACS (Table 1)

**Table 1:** Studies on the impacts of NAFLD on the development of ACS

Author/year/country	Study design and population	NAFLD diagnosis	FU	Endpoint	Key findings
Studies using imaging techniques for NAFLD diagnosis					
Hamaguchi, 2007, Japan [25]	Prospective observational cohort, 1221 healthy participants (231 NAFLD and 990 non-NAFLD)	US	7115 person-years	CAD (UA, AMI and silent MI), ischemic stroke, and cerebral hemorrhage	NAFLD was an independent predictor of CVD (OR 4.12, 95% CI 1.58–10.75)
Fracanzani, 2016, Italy [125]	Prospective matched cohort, 125 NAFLD and 250 matched controls	US and histology	10 yrs	ACS, cardiac catheterization and revascularization, cerebral ischemic stroke, and TIA	NAFLD had an increased risk of developing major cardiovascular events (HR 1.99, 95% CI 1.01–3.94)
Zeb, 2016, USA [26]	Post-hoc analysis of the MESA study, 6814 participants free of clinical CVD	unenhanced CT	7.6 yrs	Nonfatal CAD (MI, angina and revascularization) and all-cause mortality events	NAFLD was independently associated with incident nonfatal CAD and all-cause mortality events (HR 1.42, 95% CI 1.00–2.03)
Lin, 2021, USA [27]	Post-hoc analysis of the prospective EISNER cohort study, 2068 symptomatic individuals	unenhanced CT	14 yrs	MACE (MI, late revascularization, or cardiac death)	NAFLD predicted MACE (HR 1.78, 95% CI 1.21–2.61) independently of MetS, CAC score, and EAT measures
Xu, 2021, China [126]	Prospective community-based cohort, 79,905 participants without history of stroke, cancer or MI	US	10.34 yrs	MI and combined vascular events (secondary outcomes)	Presence of NAFLD was associated with increased risk of MI (HR 1.27, 95% CI 1.12–1.44)
Pickhardt, 2014, USA [28]	Retrospective cohort, 282 steatosis and 768 controls	unenhanced CT	7 yrs	MI, CVA, documented TIAs, and CABG or stenting	Steatosis was **not** an independent risk factor for subsequent cardiovascular events after controlling for diabetes and BMI (OR 1.110, 95% CI 0.553–2.228)
Liu, 2017, China [29]	Retrospective cohort, 5848 consecutive patients	TE-CAP	26 months	liver-related events, non-HCC cancers, and CVE (ACS, CVA, stent or CABG)	Neither the presence nor the severity of hepatic steatosis as measured by CAP predict CVE in the short term (HR 0.999, 95% CI 0.994–1.003)
Targher, 2005, Italy [18]	prospective nested case–control, 2103 T2DM patients without diagnosed CVD (248 case individuals, 496 control individuals)	US	5 yrs	MI, ischemic stroke, coronary revascularization, and CV death	NAFLD was independently associated with an increased risk of future CVD events (OR 1.53, 95% CI 1.1–1.7)
Targher, 2007, Italy [19]	case–control, 2103 T2DM patients without diagnosed CVD (384 case individuals, 1719 control individuals)	US	6.5 yrs	MI, ischemic stroke, coronary revascularization, and CV death	NAFLD was independently associated with an increased incidence of CVD events (HR 1.87, 95% CI 1.2–2.6)
Wong, 2011, China [24]	prospective cohort, 612 patients with clinical indications for CAG (356 fatty liver and 256 no fatty liver)	US	87 ± 22 weeks	CV deaths, non-fatal MI, and the need for coronary intervention	Fatty liver could **not** predict adverse cardiovascular outcomes in patients with established CAD (HR 0.93, 95% CI 0.52–1.66)
Meyersohn et al. 2021 [21]	nested cohort, 3756 outpatients with suspicion for OCAD undergoing coronary CTA (2797 no hepatic steatosis and 959 hepatic steatosis)	unenhanced CT	25 months	death, MI, and unstable angina	Hepatic steatosis was associated with MACE independent of ASCVD risk scores, significant stenosis, and Mets (aHR 1.72, 95% CI 1.16–2.54) or obesity (aHR 1.75, 95% CI 1.19–2.59)
Database studies using ICD code for NAFLD definition					
Allen, 2019, USA [32]	Longitudinal matched cohort, 3869 NAFLD, 15,209 controls	ICD code	7 yrs	MI, angina, and stroke	Female advantage in CVD protection is lost in NAFLD individuals (HR 0.71, 95% CI 0.62–0.80 vs. HR 0.90, 95% CI 0.74–1.08)
Labenz, 2020, Germany [31]	Population-based matched cohort, 22,048 NAFLD/NASH and 22,048 controls	ICD code	10 yrs	MI, CAD, AF, and stroke	NAFLD constituted an independent risk factor for CAD, MI, and AF in primary care in Germany (HR 1.34, 95% CI 1.10–1.63)
Ghoneim, 2020, USA [30]	Nationwide population-based retrospective cohort, 55,099,280 subjects, of which 43,170 NASH	ICD code	/	AMI	NASH was associated with MI independent of traditional risk factors (OR 1.5, 95% CI 1.40–1.62)
Alexander, 2019, multi-country [33]	matched cohort, 120,795 NAFLD/NASH and up to 100 matched controls for each case	ICD code	2.1–5.5 yrs	fatal or non-fatal AMI and ischemic or unspecified stroke	NAFLD was **not** associated with AMI risk after adjustment for established cardiovascular risk factors (HR 1.01, 95% CI 0.91–1.12)
Labenz, 2021, Germany [20]	retrospective matched cohort, 2633 T2DM with NAFLD and 2633 T2DM without liver disease	ICD code	10 yrs	MI	NAFLD was **not** significantly associated with the incidence of MI (HR 0.77, 95% CI 0.58–1.03)
Studies using surrogate markers for NAFLD definition					
Seo, 2021, South Korea [127]	population-based study, 334,280 otherwise healthy NAFLD patients	FLI	5.4 yrs	a composite of cardiovascular death, MI, ischemic stroke, or coronary revascularization	A higher FLI was independently associated with an increased risk for CVD (HR between Q4 and Q1 1.92, 95% CI 1.63–2.25)
Kim, 2020, South Korea [35]	nationwide population-based cohort, 3,011,588 individuals without a history of CVD	FLI	6 yrs	a composite of CV deaths, non-fatal MI, and ischemic stroke	A higher FLI was significantly associated with an increased risk of non-fatal MI (HR between Q4 and Q1 2.16, 95% CI 2.01–2.31)
Lee, 2021, South Korea [36]	population-based cohort, 3,003,068 healthy individuals	FLI	5.1 yrs	MI, stroke, and all-cause mortality	Individuals with repeatedly elevated FLI had a higher risk of incidence of MI (aHR 1.3, 95% CI,1.21–1.40)
Chung, 2022, South Korea [37]	population-based cohort, 5,324,410 young adults aged 20 to 39 years	FLI	8.4 yrs	MI and stroke	NAFLD was an independent predictor of MI in young adults (HR 1.69, 95% CI 1.61–1.77)
Park, 2022, South Korea [38]	nationwide cohort, 139,633 patients with new-onset T2DM	FLI	7.7 yrs	MI, ischemic stroke, HF, and all-cause death	FLI ≥ 60 was significantly associated with increased risk for MI (HR 1.28, 95% CI 1.14–1.44)
Olubamwo, 2018, Finland [34]	prospective cohort, 1205 middle-aged men free from CVD	FLI	17 yrs	incident CVD and incident AMI	The predictability of AMI using FLI is subject to interactions of metabolic factors (HR 1.136, 95% CI 0.777–1.662)
Sinn, 2020, South Korea [41]	retrospective cohort, 11,492 adults over 40 years old without history of CVD, liver disease, or cancer	US, NFS, and FIB-4	725,706.9 person-years	incident MI	NAFLD was independently associated with an increased incidence of MI (HR 1.54, 95% CI 1.11–2.14)
Baratta, 2020, Italy [12]	analysis of a prospective cohort, 898 consecutive outpatients	NFS and FIB-4	41.4 months	fatal or nonfatal ischemic stroke and MI, cardiac or peripheral revascularization, new-onset AF and CV death	Individuals with NAFLD had more than a twofold increase in risk of CVEs, and those with liver fibrosis had a fourfold increase in risk
Vieira, 2022, USA [40]	real-world cohort, 67,273 patients without previous CVD	FIB-4	3 yrs	MI, hospitalization for UA or HF, and revascularization	FIB-4 ≥ 2.67 was independently associated with MI (aHR 1.46, 95% CI 1.25–1.70)
Loosen, 2022, Germany [42]	Database study, 68 921 patients with available lab values for FIB-4 score calculation	FIB-4	/	MI	FIB-4 score is **not** associated with CVE both in the general population as well as in patients with chronic liver disease
Liu, 2021, China [39]	multicenter prospective study, 4003 SCAD after elective PCI	LFS**	5.0 ± 1.6 yrs	cardiovascular death, nonfatal MI, and stroke	High LFSs levels might be useful for predicting adverse prognosis
Lee, 2021, China [128]	cross-sectional, 17,244 adults	LFS	/	MI	LFS was **not** associated with MI
Studies of MAFLD					
Lee, 2021, South Korea [16]	nationwide population-based cohort, 8,962,813 participants aged 40–64 years without prior CVD	MAFLD	10.1 yrs	a composite CVE, including MI, ischemic stroke, HF, or CVD-related death	When the Neither-FLD group was the reference, multivariable-adjusted HR (95% CI) for CVD events were 1.09 (1.03–1.15) in the NAFLD-only group, 1.43 (1.41–1.45) in the MAFLD-only group, and 1.56 (1.54–1.58) in the Both-FLD group
Moon, 2021, South Korea [43]	community-based prospective cohort, 8919 participants, in which 1509 MAFLD	MAFLD	15.7 yrs	mortality and CVD events (AMI, CAD, or cerebrovascular disease)	MAFLD independently increased overall mortality (HR 1.33, 95% CI 1.05–1.69). MAFLD also independently predicted CVD after adjustment for age, sex, and BMI (HR 1.35, 95% CI 1.13–1.62), which lost its statistical significance by further adjustments
Niriella, 2021, Sri Lanka [17]	community-based prospective cohort, 2985 individuals, of which 940 NAFLD, 990 MAFLD and 362 controls	MAFLD	7 yrs	fatal or non-fatal MI or stroke, CABG, and PTCA	Patients excluded by the NAFLD definition but captured by the MAFLD definition were at higher risk of adverse outcomes than those excluded by the MAFLD definition but captured by the NAFLD definition

US ultrasound, CAD Coronary artery disease, UA unstable angina, AMI Acute myocardial infarction, MI Myocardial infarction, NAFLD Non-alcoholic fatty liver disease, ACS Acute coronary syndrome, TIA transient ischemic attack, CT Computed tomography, MACE Major adverse cardiovascular event, CVA Cerebrovascular accident, Mets Metabolic syndrome, EAT Epicardial adipose tissue, CABG Coronary artery bypass graft, TE-CAP Transient elastography-controlled attenuation parameter, HCC Hepatocellular carcinoma, CVE cardiovascular event, T2DM Type 2 diabetes mellitus, CVD Cardiovascular disease, CAG Coronary angiography, AF Atrial fibrillation, NASH Non-alcoholic steatohepatitis, FLI Fatty liver index, NFS NAFLD fibrosis score, FIB-4 Fibrosis-4 score, MAFLD Metabolic dysfunction-associated fatty liver disease, FLD Fatty liver disease, PTCA Percutaneous transluminal coronary angioplasty, LFS Liver Fat Score, LFS** Liver Fibrosis Score

There are several findings on whether NAFLD increases the risk of ACS in specific disease populations and otherwise healthy subjects.

As early as 2005, Targher et al. conducted a prospective nested case–control study on T2DM patients [18]. This study indicated, for the first time, that NAFLD was independently associated with an increased risk of future CVD events, including non-fatal myocardial infarction (MI), coronary revascularization, ischemic stroke, and cardiovascular death (OR 1.53, 95% CI 1.1–1.7, *p* = 0.02) [18]. Subsequently, they further validated their conclusion with a longer follow-up in 2007 [19]. However, a database cohort study using the International Classification of Diseases (ICD) for subgrouping in Germany last year had different results [20]. According to this study, there was no significant association between NAFLD and the incidence of MI in T2DM individuals (HR 0.77, 95% CI 0.58–1.03, *p* = 0.08) [20]. A possible explanation for this may be the difference in endpoint selection and NAFLD diagnosis methods. In addition to T2DM, studies have been performed on the coronary artery disease (CAD) population. Some recent studies demonstrated that hepatic steatosis was independently associated with main adverse cardiovascular events (MACEs) in symptomatic patients with suspected CAD [21, 22]. Thus, concurrent evaluation of hepatic steatosis in this patient group may help to identify subjects at higher risk of MACEs [22, 23]. However, a prior study showed that although fatty liver was independently associated with significant CAD in patients with clinical indications for coronary angiography (CAG) (aOR 2.31, 95% CI 1.46–3.64, *p* < 0.001), its presence did not increase the incidence of cardiovascular deaths, non-fatal MI, or the need for revascularization in patients with established CAD (HR 0.93, 95% CI 0.52–1.66, *p* = 0.79) [24]. Apart from its small sample size, it is noteworthy that the mean follow-up time of this study was quite short (87 ± 22 weeks) [24].

The findings among healthy individuals are also divergent. A prospective observational cohort study involving apparently healthy Japanese men and women indicated that NAFLD was a predictor of cardiovascular events, including unstable angina, AMI, silent MI, ischemic stroke, and cerebral hemorrhage, independent of conventional cardiovascular risk factors (OR 4.12, 95% CI 1.58–10.75, *p* = 0.004) [25]. Moreover, post-hoc analyses of the MESA study [26] and the EISNER cohort study [27] showed that NAFLD was associated with adverse events in asymptomatic patients free of CVD. However, a longitudinal investigation using unenhanced CT failed to identify hepatic steatosis as an independent risk factor for subsequent cardiovascular events (OR 1.110, 95% CI 0.553–2.228, *p* = 0.77) [28]. Liu et al. quantified hepatic steatosis by transient elastography-controlled attenuation parameter (TE-CAP). They found that neither the presence nor the severity of hepatic steatosis predicted liver-related events, cancer, or cardiovascular events in the short term [29]. However, this study cohort was heterogeneous, so the results should be interpreted with caution. In addition, database studies have shown differential results. Ghoneim et al. reported that NASH was associated with MI independent of traditional risk factors (OR 1.5, 95% CI 1.40–1.62). Compared to the older NASH population, younger patients were more likely to have a higher relative risk of MI [30]. A population-based matched cohort in primary care patients in Germany reached similar conclusions [31]. Allen et al. confirmed this association from another angle. They found that the advantage of female sex in CVD protection was lost in NAFLD individuals (HR 0.71, 95% CI 0.62–0.80, *p* < 0.001 vs. HR 0.90, 95% CI 0.74–1.08, *p* = 0.25; general population vs. NAFLD population). Moreover, women with NAFLD developed CVD at a younger age than their counterparts in the general population [32]. However, another database study performed by Alexander et al. showed that the diagnosis of NAFLD was not associated with AMI (HR 1.01, 95% CI 0.91–1.12) or stroke risk after adjustment for established cardiovascular risk factors [33]. Discrepancies in the strength of adjustment may partly explain the different results.

In addition to imaging techniques, several non-invasive surrogates based on simple clinical, anthropometric, and laboratory data have emerged as validated markers for the detection of NAFLD. Olubamwo et al. first investigated the prospective association between fatty liver index (FLI) and the risk of incident AMI in Finland. They reported that FLI could predict incident CVD, but the predictive value of FLI for AMI was subject to interactions with metabolic factors (HR 1.136, 95% CI 0.777–1.662, *p* = 0.510) [34]. However, several studies in Asia reached different conclusions. A population-based cohort study done by Kim et al. concluded that a higher FLI was an independent predictor of the development of MI (HR 2.16, 95% CI 2.01–2.31), ischemic stroke (HR 2.01; 95% CI 1.90–2.13), and cardiovascular mortality (HR 1.98, 95% CI 1.90–2.06) [35]. There was a linear relationship between the FLI and adverse outcome measures [35]. Subsequently, some other studies in South Korea further confirmed the prognostic value of FLI for MI among healthy individuals [36], young adults [37], and patients with new-onset T2DM [38].

The NAFLD fibrosis score (NFS) and the Fibrosis-4 score (FIB-4) are the two most commonly studied scoring systems for identifying advanced NAFLD and liver fibrosis. In patients with stable CAD following percutaneous coronary intervention (PCI), a study using eight liver fibrosis scores (including NFS and FIB-4) demonstrated that higher baseline liver fibrosis scores were significantly associated with the risk of cardiovascular events [39]. Baratta et al. reported that individuals with NAFLD had a more than twofold increased risk of cardiovascular events, including MI, and those with liver fibrosis had a fourfold increased risk [12]. A real-world cohort further confirmed FIB-4 score ≥ 2.67 as an independent predictor of MACEs (aHR 1.80, 95% CI 1.61–2.02, *p* < 0.001) and MI (aHR 1.46, 95% CI 1.25–1.70) beyond the established cardiovascular risk factors and NAFLD stage at baseline [40]. However, it is interesting to note that two other investigations using MI as the single endpoint yielded different results. Sinn et al. demonstrated that NAFLD was associated with an increased incidence of MI independent of established risk factors (HR 1.54, 95% CI 1.11–2.14), but this association was not related to the presence or absence of more advanced NAFLD indicated by NFS [41]. Another study found that an elevated FIB-4 score was not associated with MI incidence in either the general population or patients with chronic liver disease [42].

The term MAFLD has recently been suggested as an alternative for NAFLD, as it better reflects the metabolic dysfunction. A prospective, community-based cohort study with median follow-up of 15.7 years indicated that MAFLD independently predicted CVD after adjustment for age, sex, and body mass index (HR 1.35, 95% CI 1.13–1.62, *p* = 0.001), but the statistical significance was lost after further adjustments [43]. Similar findings were obtained in two other studies using MAFLD diagnosis [16, 17].

### NAFLD and Coronary Severity in ACS (Table 2)

**Table 2:** Studies on the impacts of NAFLD on coronary severity and outcomes in ACS

Author/year/country	Study design	Population	NAFLD diagnosis	Key findings
Agaç, 2013, Turkey [49]	cross-sectional study	80 ACS	US	The presence and stage of NAFLD was significantly correlated with CAD complexity as assessed by SYNTAX Score
Perera, 2016, Sri Lanka [51]	descriptive study	120 non-fatal ACS	US	Patients with NAFLD had a higher mortality from ACS both during hospitalization (aOR 31.3; 95% CI 2.2–439.8) and at 6 months after discharge (aOR 15.59; 95% CI 1.6–130.6)
Ravichandran, 2012, Canada [53]	retrospective cohort	528 ACS	ALT as a marker of NAFLD	An elevated ALT > 90th percentile was significantly associated with all-cause mortality in-hospital and up to 6 months after discharge (aOR 8.96, 95% CI 3.28–24.49)
Montemezzo, 2020, Brazil [45]	cross-sectional study	139 ACS undergoing diagnostic CAG	US	The intensity of NAFLD measured by US is strongly associated with the severity of coronary artery obstruction on CAG (p < 0.001)
Noda, 2022, Japan [54]	retrospective observational study	479 ACS	MAFLD	The combination of MAFLD and lower physical function independently predicted poor prognosis in ACS
Boddi, 2013, Italy [47]	observational study	95 non-DM STEMI undergoing PPCI	US	Worsening grades of NAFLD were associated with a threefold risk for multivessel CAD
Emre, 2015, Turkey [48]	prospective cohort	186 non-DM STEMI undergoing PPCI	US	FLD score ≥ 3 was an independent predictor of in-hospital MACE (OR 2.454, 95% CI 1.072–4.872)
Keskin, 2017, Turkey [50]	retrospective observational study	360 STEMI undergoing PPCI	US	NAFLD and its grade had an independent effect on both unfavorable in-hospital (OR 4.0, 95% CI 3.0–8.1) and long-term clinical outcomes (HR 4.0, 95% CI 2.4–10.9) as well as CAD severity assessed by the SYNTAX score (*p* < 0.001)
Xia, 2020, China [46]	observational study	325 elderly AMI	US	NAFLD was an independent predictor for adverse cardiovascular events (OR 1.112, 95% CI 1.043–1.324)
Simon, 2018, multi-country [52]	analysis of an international RCT	14,819 post-ACS	NFS as a marker of NAFLD	Patients in the high-risk NFS group (NFS > 0.67) had a 30% higher risk of CV events comparing to the low-risk NFS group (NFS < -1.455) (HR 1.30, 95% CI 1.19–1.43)
Öztürk, 2016, Turke y[44]	unclear	224 MI, SCAD and non-CAD	US	The severity of CAD assessed by Gensini score was significantly correlated to hepatosteatosis grade (*r* = 0.648, *p* < 0.001)
Wong, 2016, China [55]	prospective cohort	612 patients requiring CAG, including ACS, stable angina, valvular disease and others	US	The presence of NAFLD did **not** increase mortality or cardiovascular complications (aHR 0.90, 95% CI 0.69–1.18)
Ali, 2021, USA [56]	retrospective cross-sectional	429,855 patients undergoing PCI	ICD code	NAFLD did **not** increase in-hospital mortality (aOR 1.11, 95% CI 0.570–2.164) or MACE (aOR 1.115, 95% CI 0.653–1.905)

US ultrasound, MI Myocardial infarction, NAFLD Non-alcoholic fatty liver disease, ACS Acute coronary syndrome, MACE Major adverse cardiovascular event, DM Diabetes mellitus, CAG Coronary angiography, FLI Fatty liver index, NFS NAFLD fibrosis score, MAFLD Metabolic dysfunction-associated fatty liver disease, AMI Acute myocardial infarction, SCAD Stable coronary artery disease, ALT Alanine aminotransferase, STEMI ST-segment elevation myocardial infarction, PCIPercutaneous coronary intervention

Multiple studies have demonstrated that NAFLD is associated with coronary stenosis severity and complexity in ACS patients.

A study by Öztürk et al. found that NAFLD was more prevalent in MI patients than in stable CAD patients. In addition, a significant correlation between the severity of CAD as assessed by the Gensini score and hepatosteatosis grade was found in this study (*r* = 0.648, *p* < 0.001) [44]. A cross-sectional analysis by Montemezzo et al. demonstrated that the intensity of NAFLD measured by ultrasound was strongly associated with the severity of coronary artery obstruction on angiography among patients with ACS (*p* < 0.001) [45]. Similar findings were obtained in a study including 325 elderly AMI patients. The proportion of patients undergoing coronary artery bypass graft (CABG) or PCI was remarkably higher in the NAFLD group than in the non-NAFLD group (34% vs. 16%, *p* < 0.001), indicating an association between NAFLD and coronary artery stenosis severity [46].

Additionally, multivessel and complex coronary diseases are also frequently observed in ACS patients with NAFLD. Boddi et al. found a very high prevalence of NAFLD in nondiabetic patients admitted for STEMI. Worsening grades of NAFLD were associated with a threefold risk for multivessel CAD associated with cardiovascular events and this association remained significant after adjustment for waist circumference and age [47]. Emre et al. conducted a study in non-diabetic patients who underwent primary PCI for STEMI and found that multivessel coronary disease was more common in the moderate-to-severe NAFLD group (FLD score ≥ 3) than the mild group (FLD score < 3) (72% vs. 51%, *p* = 0.003). Moreover, abnormal myocardial perfusion was also significantly more frequent in patients with moderate-to-severe NAFLD [48]. Previous research has also demonstrated that ACS patients with NAFLD have more complex CAD as assessed by the SYNTAX score than those without NAFLD (18 ± 8 vs. 11 ± 5, *p* = 0.001), and the stage measured by ultrasound was significantly associated with the SYNTAX score in univariate analysis (*r* = 0.6, *p* < 0.001) [49]. In addition, further multivariate logistic analysis revealed that the presence of NAFLD was an independent factor associated with the supra-median SYNTAX score (OR 13.20, 95% CI 2.52–69.15, *p* = 0.002) [49]. Similar results for the SYNTAX score were obtained in another observational study involving 360 STEMI patients [50].

### NAFLD and Clinical Outcomes in ACS (Table 2)

The association between NAFLD and CVD has been well-established by substantial evidence. However, study results concerning the association of NAFLD and cardiovascular outcomes in ACS patients remain discordant.

A study involving 120 non-fatal ACS patients demonstrated that the ultrasound-determined NAFLD group had a significantly higher GRACE score (120.2 ± 26.9 vs. 92.3 ± 24.2, *p* < 0.001) and exhibited higher predicted mortality from ACS both during hospitalization (aOR 31.3, 95% CI 2.2–439.8, *p* = 0.011) and at 6 months after discharge (aOR 15.59, 95% CI 1.6–130.6, *p* = 0.011), thus requiring aggressive treatment of CAD [51]. In addition, two studies using surrogates of NAFLD have reached similar conclusions. Simon et al. applied NFS to 14,819 post-ACS patients from the IMPROVE-IT trial population. They concluded that the high-risk NFS group (NFS > 0.67) had a 30% higher risk of cardiovascular events than the low-risk NFS group (NFS < –1.455) (HR 1.30, 95% CI 1.19–1.43, *p* < 0.001) [52]. The GRACE-ALT study found that NAFLD, diagnosed by elevated serum alanine aminotransferase (ALT), was significantly associated with more cardiac muscle injury (aOR 7.07, 95% CI 1.83–27.37) and all-cause mortality in-hospital and up to 6 months after discharge among ACS patients (aOR 8.96, 95% CI 3.28–24.49) [53]. However, these results may be biased, as ALT is also one of the myocardial enzymes representing myocardial injury. Recently, with the introduction of MAFLD as a new name for NAFLD, Noda et al. investigated the relationship between MAFLD and physical dysfunction and prognosis in 479 hospitalized ACS patients. They demonstrated that MAFLD was independently associated with lower leg strength, gait speed, and 6-min walking distance (6MWD) (*p* = 0.020; *p* = 0.003; *p* = 0.011, respectively). Furthermore, the combination of MAFLD and reduced physical functions was an independent predictor of adverse outcomes in ACS patients, after a median follow-up period of 1.43 years [54].

Similar findings were obtained in some studies of the STEMI subtype. Emre et al. conducted a prospectively designed cohort analysis of 186 nondiabetic patients who underwent primary PCI for STEMI and found that patients with moderate-to-severe NAFLD were more likely to have in-hospital MACE (31% vs. 8%, *p* < 0.0001), defined as nonfatal MI, acute heart failure (HF), and death. An FLD score ≥ 3 was found to be an independent predictor of in-hospital adverse outcomes (OR 2.454, 95% CI 1.072–4.872, *p* = 0.048) using multivariate analysis [48]. A retrospective observational study involving 360 STEMI patients showed that in-hospital mortality for patients with grade 0, 1, 2, and 3 NAFLD was 4.7%, 8.3%, 11.3%, and 33.9%, respectively. The three-year mortality rates for these groups were 5.6%, 7.8%, 9.5%, and 33.3%, respectively [50]. Among these patients, grade 3 NAFLD was the main subgroup with higher rates of stent thrombosis and mortality, indicating that the presence of NAFLD was associated with unfavorable clinical outcomes in patients with STEMI [50]. Another study reported that elderly MI patients with NAFLD had a significantly higher risk for adverse cardiovascular events than those without NAFLD, including ECG instability (26% vs. 15%, p < 0.001), hemodynamic instability (33% vs. 14%, *p* = 0.033), and death during hospitalization (7% vs. 5%, *p* = 0.016). Moreover, a GRACE score > 140 (OR 3.005, 95% CI 1.504–6.032, *p* = 0.002), EF < 35% (OR 2.649, 95% CI 1.364–4.346, *p* = 0.009), diabetes (OR 1.308, 95% CI 1.072–1.589, *p* = 0.015), and NAFLD (OR 1.112, 95% CI 1.043–1.324, *p* = 0.024) were independent predictors for adverse cardiovascular events in these patients [46].

However, some studies have opposed the association between NAFLD and adverse outcomes in ACS patients. A prior study involved 612 patients with indications for CAG, including ACS, stable angina, valvular disease, and others [55]. Wong et al. found that the presence of NAFLD was associated with coronary artery stenosis and the need for coronary intervention, but it did not increase composite cardiovascular outcomes (cardiovascular death, non-fatal MI, HF, or the need for further interventions) (37.1% vs. 36.5%, aHR 0.90, 95% CI 0.69–1.18, *p* = 0.46) in patients with indications for CAG after 3679 patient-years of follow-up [55]. Another study conducted by Ali et al. recently divided 429,855 patients undergoing PCI into NAFLD and non-NAFLD groups [56]. These two groups had similar proportions of patients presenting with NSTEMI and STEMI. They found that patients with NAFLD had a longer length of hospital stay, were admitted at a younger age, and had significantly more cardiovascular comorbidities, while no difference was found for in-hospital mortality between these two groups (aOR 1.11, 95% CI 0.570–2.164, *p* = 0.757) [56]. A possible explanation for the discrepant conclusions may be the composition and distribution of patients in these two studies, as the inclusion of non-ACS individuals in the studies may somewhat attenuate the differences in prognosis between the groups.

## Potential Mechanisms of NAFLD in ACS (Fig. 1)

**Fig. 1:**
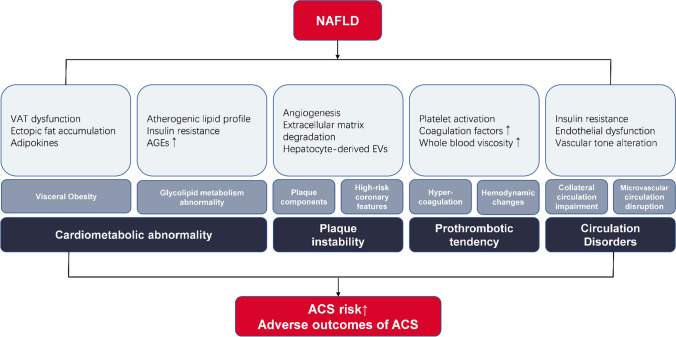
Potential mechanisms of NAFLD in ACS. NAFLD: non-alcoholic fatty liver disease; ACS: acute coronary syndrome; VAT: visceral adipose tissue; AGEs: advanced glycation end products; EVs: extracellular vesicles.

Systemic inflammation, endothelial dysfunction, hepatic insulin resistance, oxidative stress, and altered lipid metabolism are some of the mechanisms by which NAFLD increases the risk of CVD [13]. Currently, there is a considerable body of research suggesting that NAFLD may increase ACS risk and lead to adverse outcomes in ACS patients, although the results are somewhat controversial. The exact pathophysiological mechanisms underlying this complex association between NAFLD and ACS remain unidentified, and few studies have directly addressed this issue.

### Cardiometabolic Factors

The liver plays a crucial role in lipid and glucose metabolism and therefore is at the center of cardiometabolic disease.

Several studies reported that ACS patients with NAFLD had a significantly higher body mass index (BMI) and waist circumference, diabetes or metabolic syndrome, and increased levels of serum TGs and low-density lipoprotein cholesterol (LDL-c) than those without NAFLD, while their high-density lipoprotein cholesterol (HDL-c) was significantly lower [46–49, 51]. Similar findings were obtained in a recent study investigating MAFLD in hospitalized ACS patients. Younger age, obesity, dyslipidemia, higher BMI, waist circumference, fatty liver index (FLI), and poor liver function (ALT, AST, and γ-GTP) were more likely to be observed in patients with MAFLD [54].

#### Visceral Obesity

Expanded visceral adipose tissue (VAT) has an elevated expression of proinflammatory mediators in NAFLD/NASH patients, and adipocyte tissue macrophages produce increased levels of inflammatory cytokines, thus leading to low-grade systemic inflammation [57]. It has been demonstrated that coronary plaque vulnerability appears to be more closely related to abnormal abdominal fat distribution than with visceral or subcutaneous fat amount alone in patients with ACS [58].

In addition to dysfunctional VAT, emerging evidence has suggested that an accumulation of ectopic fat in the liver, epicardium, pancreas, kidneys or muscle might contribute to increased atherosclerotic and cardiometabolic risk [59, 60]. Epicardial adipose tissue (EAT) shares an unobstructed microcirculation with the adjacent myocardium due to its anatomical intimacy with the heart [61]. A prior meta-analysis indicated that an increase in EAT was associated with the severity of steatosis, fibrosis, and CVD in patients with NAFLD [62]. EAT is also thought to be a robust measure of increased risk of MI [63]. With increasing EAT volume, dangerous plaque composition burdens and high-risk features increase significantly, which could be associated with unfavorable outcomes in ACS patients [64, 65].

Moreover, adipose tissue is far more than a simple fat storage depot. Adipokines, peptides produced by white adipose tissue, are active metabolic players implicated in both NAFLD and CVD pathogenesis with autocrine, paracrine, and endocrine functions [66]. Adiponectin exerts beneficial systemic metabolic effects through its actions on adipogenesis, atherosclerosis, insulin sensitivity, and inflammation, while elevated levels of leptin are associated with obesity, leptin resistance, and adverse effects on the heart [67, 68]. Alterations in circulating adipokines, namely a decrease in adiponectin and an increase in leptin, have been observed in patients with NAFLD [67, 69].

#### Glycolipid Metabolism Abnormality

The liver plays a central role in lipid metabolism. The atherogenic lipid profile, characterized by hypertriglyceridemia, increased small dense low-density lipoprotein (sdLDL) particles and decreased HDL-c levels, is frequently observed in NAFLD patients and promotes the development of atherosclerotic plaques [70]. A predominance of sdLDL particles, as well as an accumulation of triglyceride-rich lipoproteins (TRLs), TRL remnants and intermediate-density lipoprotein (IDL), are hallmarks of the atherogenic lipoprotein phenotype [11]. This altered composition of serum lipoprotein is thought to drive atherosclerotic risk. A study by Duran et al. revealed that TRLs and sdLDL were strongly associated with future MI events in primary prevention populations [71].

Insulin resistance (IR) is strongly related to NAFLD, and it has been implicated in both the pathogenesis and disease progression of NAFLD. The prevalence of NAFLD in diabetic patients is fivefold higher than that in nondiabetic patients [72]. Insulin resistance, as well as impaired insulin signaling, is also associated with atherogenesis, atherosclerotic lesion progression, and plaque vulnerability [11, 73]. An analysis from the PROSPECT study showed that IR was common among ACS patients and that advanced stages of IR were independently associated with an increased risk of MACEs [74]. In addition, NAFLD-related steatosis is associated with serum advanced glycation end products (AGEs) levels, which induce tissue inflammation and oxidative damage [75].

### Plaque Instability

Beyond the degree of luminal stenosis, coronary plaque formation and characteristics play a crucial role in the development of CAD, especially ACS [76]. To date, there are no study data on the relationship between NAFLD and more advanced coronary plaque characteristics in ACS patients, despite the established association between NAFLD and atherosclerosis. We postulate that these findings from a more general CAD population might be consistent and referential since atherosclerosis is a continuous process.

Clinically, mixed plaques on coronary CTA were thought to be associated with the worst prognosis, calcified plaques were associated with the best event-free survival, and noncalcified plaques had an associated risk in between the other two types [77]. Kang et al. demonstrated that NAFLD was associated with the presence and calcific morphology of coronary atherosclerotic plaques in asymptomatic individuals without a history of CVD using multidetector computed tomography (MDCT) coronary angiography [78]. However, investigations performed by Lee et al. and Park et al. a few years later reached different conclusions. They found that NAFLD was independently associated with the presence of non-calcified plaque morphology [79, 80]. Recently, Meyersohn et al. found that mixed plaques were more frequently observed in patients with hepatic steatosis than in those without it (57.8% vs. 53.6%, *p* = 0.027) [21].

In terms of high-risk coronary features in NAFLD patients, research findings were quite consistent, suggesting an association between NAFLD and the potential plaque vulnerability in coronary arteries. According to the Coronary Artery Disease-Reporting and Data System (CAD-RADS), vulnerable plaque characteristics identified by coronary CTA include low-attenuation plaques, spotty calcification, positive remodeling, and the napkin-ring sign [81]. An early study revealed that there was a statistically significant difference between lipid pool plaques and positive remodeling in patients with NAFLD compared with those without NAFLD, whereas no statistical significance was found for the degree of coronary luminal stenosis [82]. Recent research in the general population showed that an increase in steatosis severity of NAFLD correlated with a higher coronary artery atherosclerosis burden and the presence of high-risk mixed type plaques and was independent of traditional risk factors [83•]. Saraya et al. found significant associations between NAFLD and high-risk plaque features, including the napkin ring sign (OR 7.88, *p* < 0.001), positive remodeling (OR 5.84, *p* < 0.001), low HU (OR 7.25, *p* < 0.001), and spotty calcium (OR 6.66, *p* < 0.001) in CAD patients [84]. Another study, as one arm of the randomized controlled trial (ROMICATII), found that advanced high-risk coronary plaque features were more frequently observed in patients with NAFLD and the association between them (OR 2.13, 95% CI 1.18–3.85, *p* = 0.012) was independent of the extent and severity of coronary atherosclerosis as well as traditional cardiovascular risk factors [85].

Vascular endothelial growth factors (VEGFs) have been discovered to be key mediators of angiogenesis and play an active role in the growth and destabilization of atherosclerotic lesions [86, 87]. Elevated serum VEGF levels are observed in NAFLD patients and may be a plausible explanation for plaque vulnerability [88]. Matrix metalloproteinases (MMPs) have been found to be associated with fibrosis and disease progression in NAFLD/NASH [89, 90], which could enhance matrix dissolution and promote atherosclerotic plaque instability [91]. Another aspect that may influence coronary plaque instability in NAFLD patients is the extracellular vesicles (EVs) released by hepatocytes. A study found that steatotic hepatocyte-derived EVs can promote endothelial inflammation and facilitate atherogenesis via microRNA-1 release, Kruppel-like factor 4 (KLF4) suppression, and nuclear factor-κB (NF-κB) activation [92].

Currently, the identification of coronary plaque morphology in NAFLD patients is based on coronary CTA, while intravascular imaging modalities with a higher resolution, such as intravascular ultrasound (IVUS) and optical coherence tomography (OCT), may add more details to plaque composition and characteristics. Thus, further research is needed in this field.

### Prothrombotic Tendency

Drastic consequences of thrombotic complications account for the majority of ACS cases. Accumulating evidence indicates that NAFLD is closely associated with a prothrombotic tendency and hypercoagulable state, which may to some extent explain the increased risk of ACS among NAFLD patients [93•]. A previous study showed that elevated serum homocysteine levels are common in NAFLD patients, and hyperhomocysteinemia may lead to oxidative stress and further enhance platelet activation [13, 94]. A pilot study conducted by Gidaro et al. found that patients with NAFLD had elevated levels of coagulation factors, suggesting an increased risk of thrombosis [95]. This study also suggested that systemic inflammation predisposed patients to endothelial dysfunction and thrombosis [95]. Another study indicated that liver fat played a crucial role in the regulation of protein C and protein S. It also demonstrated that NAFLD patients had elevated levels of C-reactive protein (CRP), fibrinogen, plasminogen activator inhibitor-1 (PAI-1), von Willebrand factors, and coagulation factor VII, which are known to be associated with an increased risk of thrombosis [96]. The activities of coagulation factors VIII, IX, XI, and XII activities were increased in NAFLD patients and were positively correlated with hepatic fat content, suggesting that fatty liver may contribute to the risk of thrombosis [97]. Procoagulant imbalance, resulting from the increased level of factor VIII and reduced protein C activity, was observed in patients with NAFLD [98]. In addition, it has been proven that NAFLD severity, as assessed by liver biopsy, contributes to the increase in PAI-1 levels independent of anthropometric and metabolic parameters [99]. Previous studies have noted that ACS patients with elevated whole blood viscosity are at higher risk for arterial thrombosis, acute stent thrombosis, and left ventricular apical thrombosis [100]. Whole blood viscosity at low shear stress was increased in NAFLD patients [101]. Altered hemorheological parameters may also explain the association between NAFLD and ACS to some extent.

### Circulation Disorders

Coronary collateral circulation is an important protective mechanism for cardiomyocytes in the event of occlusion in any coronary artery in ACS. A study conducted by Arslan et al. found that NAFLD was independently associated with poor coronary collateral development in nondiabetic patients with severe CAD, which may partially explain the poor prognosis of ACS patients with NAFLD [102]. Previous research demonstrated that the presence of metabolic syndrome and associated insulin resistance played an important role in impaired collateral circulation [103]. According to an animal study, the receptor for advanced glycation end products (AGEs) impaired collateral formation due to increased levels of AGEs both in diabetic and nondiabetic conditions [104].

Microvascular circulation disorder may also be a contributor. Coronary flow reserve (CFR) is widely used to evaluate the integrity of coronary microvascular circulation. A study by Yilmaz et al. indicated that CFR is impaired in patients with biopsy-confirmed NAFLD and that liver fibrosis scores are an independent predictor of depressed CFR [105]. The presence of fatty liver disease was also reported to be associated with a reduction in myocardial perfusion reserve (MPR) on MRI, suggesting impaired coronary microcirculation [106]. In addition, another study found that coronary microvascular dysfunction assessed by myocardial perfusion PET/CT was more prevalent in patients with NAFLD than in those without NAFLD and predicted MACE independently [107]. The exact underlying mechanisms are still unknown. Endothelial dysfunction and vascular tone alteration could be plausible explanations, as they are implicated in the pathophysiological mechanisms linking NAFLD and impaired microcirculation [13, 108].

## Controversies Regarding the Effects of NAFLD on ACS (Fig. 2)

**Fig. 2:**
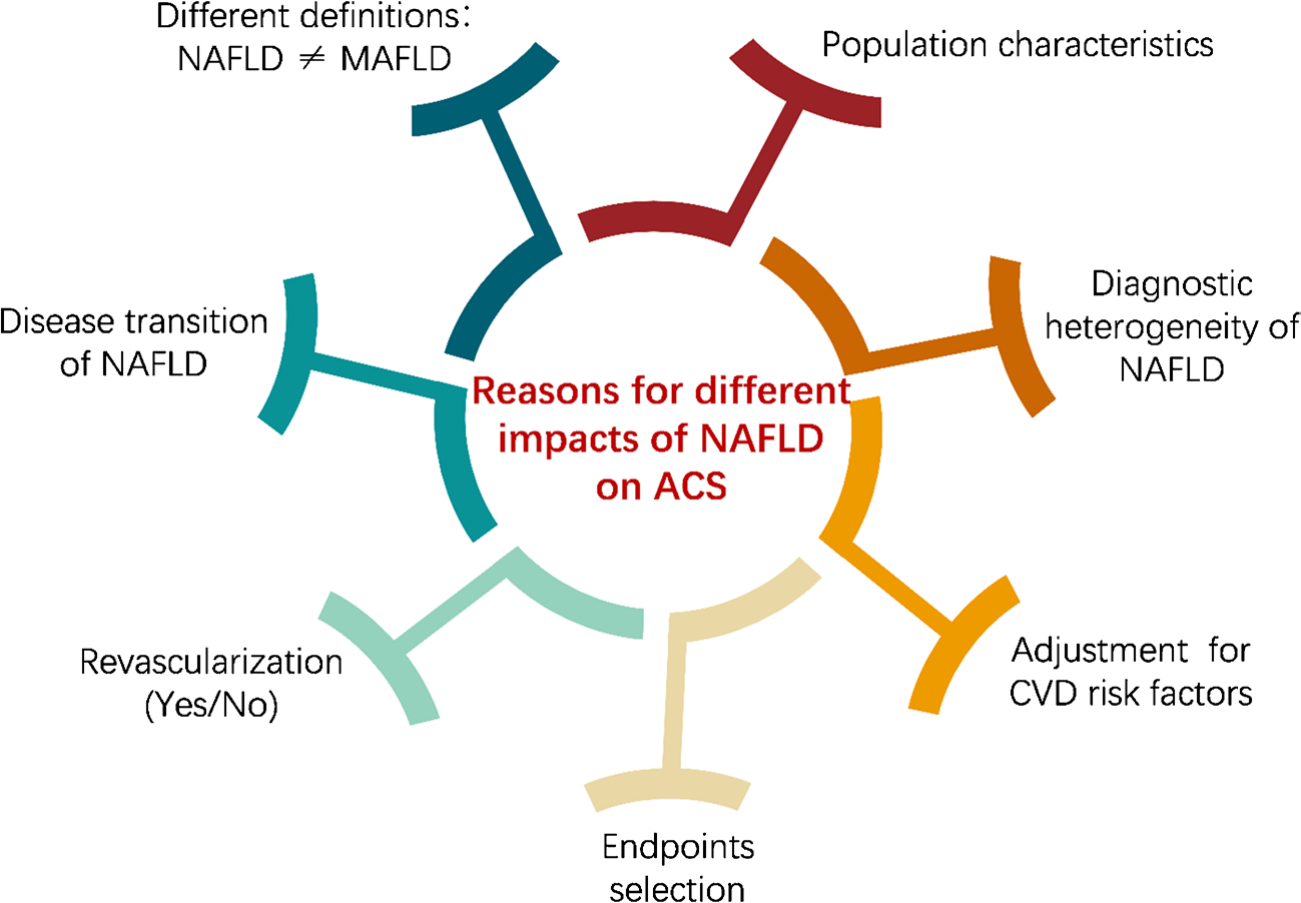
Heterogeneity of current studies on the impacts of NAFLD on ACS development and outcome. NAFLD: non-alcoholic fatty liver disease; MAFLD: metabolic dysfunction-associated fatty liver disease; ACS: acute coronary syndrome; CVD: cardiovascular disease

Although an association between NAFLD/MAFLD and ACS has been suggested, the strength of this relationship remains controversial. The disagreements mainly concentrate on whether NAFLD independently increases ACS risk and leads to more adverse outcomes in ACS patients. We conjecture that the plausible explanations for the inconsistent results of these studies are as follows.

First, baseline population characteristics must be taken into account. Patients from different racial backgrounds have varying dietary habits, lifestyles, and genetic traits, which should not be ignored. Senescence is a major risk factor for a wide range of diseases. Along with aging, cardiovascular risk factors are continuously accumulating, which may weaken the connection between NAFLD and ACS, leading to negative findings. Differences in the proportions of comorbidities, such as T2DM and dyslipidemia, can also account for divergent results.

Second, NAFLD is an exclusive diagnosis, and sometimes its etiological identification might be misleading, as alcohol consumption is not the only factor we should consider. The wide disease spectrum of this clinical entity leads to a great degree of heterogeneity in NAFLD severity. The vast majority of these studies lack histologic data owing to the invasive nature of liver biopsy. Studies in more general CVD populations previously demonstrated that while probably all subtypes of NAFLD were associated with increased cardiovascular risk, the strongest links were in patients with NASH and an advanced stage of fibrosis [109, 110]

Third, NAFLD and metabolic syndrome are closely intertwined and their effects on atherosclerosis might partially overlap [111••]. The degree of adjustment for traditional or non-traditional CVD risk factors varies among studies. Moreover, FLI and NFS, which comprise known cardiovascular risk factors (age, BMI, triglyceride levels, and waist circumference), were used as the surrogates of NAFLD in several studies. Thus, we must interpret these findings with caution.

Fourth, the selection of endpoints differed in these studies. In addition, to increase statistical efficiency, composite primary endpoints were frequently used, which may complicate and challenge the interpretation of the role of NAFLD on individual components of the composite outcome.

Fifth, ACS patients with NAFLD tended to have more severe and complex lesions in some studies, leading to a higher proportion of CABG/PCI. Notably, these intervention methods may alter the natural course of ACS and prevent adverse cardiovascular outcomes in the future, thereby resulting in negative conclusions.

Sixth, NAFLD is a dynamic state that may change according to factors such as age, medication, and disease progression. Hepatic steatosis tends to regress when it progresses to the stage of advanced fibrosis and cirrhosis [112].

Finally, MAFLD, a novel concept proposed in recent years, was demonstrated to be more practical for identifying fatty liver disease patients at high risk of disease than NAFLD in the real world [113]. Several studies involving MAFLD patients are discussed in our review. However, it is important to note that these two diagnoses are not entirely equivalent and should not be used interchangeably.

Despite the current controversial findings, we believe that it is of great clinical significance to study the association between NAFLD/MAFLD and ACS. In the future, more interventional studies, RCTs, and animal studies with rigorous designs and larger sample sizes are needed. It is also urgent to develop unified diagnostic and evaluation criteria for NAFLD.

## Clinical Implications

International guidelines recommend that all patients with NAFLD should be regularly screened for cardiometabolic abnormalities, such as obesity, T2DM, dyslipidemia, and hypertension [114, 115]. Moreover, an individualized, holistic, and multidisciplinary approach to these patients is increasingly necessary [116]. Both ACS and NAFLD are clinically prevalent disease entities and share numerous risk factors, making it of great clinical significance to investigate the relationship between them.

On the one hand, diagnosing or staging NAFLD may contribute to ACS prediction and identifying patients at high risk of ACS for early intervention. Conventional cardiovascular risk assessment models do not take insulin resistance, a condition that is closely associated with NAFLD, into account. The Framingham risk score may underestimate the cardiovascular risk in patients with metabolic dysfunction, leaving this part of the population neither treated nor closely followed up. Ichikawa et al. reported that concurrent evaluation of hepatic steatosis in patients with suspected stable CAD during coronary CTA enables more accurate detection of patients at higher risk of MACEs (a composite of cardiac death, ACS, and late revascularization). By adding hepatic steatosis to the Framingham risk score and adverse AL findings, the global χ2 score and C-statistic significantly increased from 29.0 to 49.5 (*p* < 0.001) and 0.74 to 0.81 (*p* = 0.026), respectively [22]. Similar conclusions were drawn in another study involving T2DM outpatients from this research team. They found that in addition to the coronary artery calcium score (CACS) and Framingham risk score, NAFLD assessed by CT showed incremental prognostic value for cardiovascular events. The global χ2 score and C-statistic significantly increased from 27.0 to 49.7 (*p* < 0.001) and 0.71 to 0.80 (*p* = 0.005), respectively [23].

On the other hand, treatment for NAFLD or hepatic steatosis may be expected to help reduce the incidence and improve the prognosis of ACS. Breakthroughs have been made in drug repurposing as well as extensive crosstalk between cardiovascular and other systems in recent years. Currently, there is no approved pharmacological therapy for NAFLD. Lifestyle modifications including dietary change, physical exercise, and weight loss remain the cornerstone of NAFLD management [117]. However, several potential agents for the prevention and treatment of both hepatic and extra-hepatic complications are under active investigation. The insulin sensitizer pioglitazone is a selective peroxisome proliferator-activated receptor (PPAR)-γ agonist, that exerts cardiovascular benefits by decreasing the composite outcome of all-cause mortality, non-fatal MI, or stroke in T2DM patients [118]. A meta-analysis involving eight RCTs showed that pioglitazone use improved advanced fibrosis in NASH, even in individuals without diabetes [119]. Liraglutide, a glucagon-like peptide-1 receptor agonist (GLP-1RA), was reported to reduce cardiovascular events among patients with T2DM [120] and lead to histological resolution of biopsy-proven NASH [121]. In addition to beneficial effects on CVD and reduction in LDL-c levels, proprotein convertase subtilisin/kexin type 9 inhibitor (PCSK9-i) appeared to ameliorate NAFLD/NASH [122]. An observational study demonstrated that PCSK9-i therapy significantly ameliorates steatosis biomarkers (such as the triglyceride-glucose index (TyG) and hepatic steatosis index (HSI)) in familial hypercholesterolemia (FH) patients with low TG/HDL levels [123]. Obeticholic acid, a selective farnesoid X receptor agonist, is one of the most promising medications for NASH under investigation. Owing to its adverse effect on the lipid profile, it should be carefully considered in CVD patients [124]. Overall, however, there is no direct evidence that drugs targeting NAFLD reduce the incidence of cardiovascular disease and improve its prognosis.

## Conclusion

Clinically, a growing number of patients with young age, female gender, and few traditional cardiovascular risk factors are afflicted with ACS. Meanwhile, coronary plaques are biologically characterized by a gradual increase in the proportion of plaque erosion compared with plaque rupture. Cardiometabolic disorders such as NAFLD have attracted extensive attention in the cardiovascular field. NAFLD may be part of a vast network of metabolic abnormalities with multiple bidirectional relationships. It is safe to say that NAFLD is closely associated with cardiovascular disease, especially coronary artery disease. However, current research findings are still controversial concerning the impacts of NAFLD on ACS. Future larger-scale, prospective studies may be needed to examine the association between NAFLD and the incidence and outcomes of ACS. Intensive mechanistic studies are also warranted to elucidate its clinical implications, which could contribute to a better understanding of the relationship between NAFLD and ACS.

## Data Availability

Not applicable.

## Abbreviations

*6MWD*: 6-Minute walking distance
*95%CI*: 95% Confidence interval
*ACS*: Acute coronary syndrome
*AF *: Atrial fibrillation
*AGEs*: Advanced glycation end products
*aHR*: Adjusted HR
*ALT*: Alanine aminotransferase
*AMI*: Acute myocardial infarction
*aOR*: Adjusted odds ratio
*AST*: Aspartate aminotransferase
*BMI*: Body mass index
*CABG*: Coronary artery bypass graft
*CACS*: Coronary artery calcium score
*CACS*: Coronary artery calcium score
*CAD*: Coronary artery disease
*CAD-RADS*: Coronary Artery Disease-Reporting and Data System
*CAG*: Coronary angiography
*CAP*: Controlled attenuation parameter
*CFR*: Coronary flow reserve
*CRP*: C-reactive protein
*CT*: Computed tomography
*CTA*: Computed tomography angiography
*CVA*: Cerebrovascular accident
*CVD*: Cardiovascular disease
*CVE*: Cardiovascular event
*EAT*: Epicardial adipose tissue
*ECG*: Electrocardiogram
*EF*: Ejection fraction
*EV*: Extracellular vesicle
*FH*: Familial hypercholesterolemia
*FIB-4*: Fibrosis-4 score
*FLD*: Fatty liver disease
*FLI*: Fatty liver index
*GLP-1RA*: Glucagon-like peptide-1 receptor agonist
*HCC*: Hepatocellular carcinoma
*HDL-c*: High-density lipoprotein cholesterol
*HF*: Heart failure
*HR*: Hazard ratio
*HSI*: Hepatic steatosis index
*ICD*: International Classification of Diseases
*IDL*: Intermediate-density lipoprotein
*IR*: Insulin resistance
*IVUS*: Intravascular ultrasound
*KLF4*: Kruppel-like factor 4
*LDL-c*: Low-density lipoprotein cholesterol
*MACE*: Major adverse cardiovascular event
*MAFLD*: Metabolic dysfunction-associated fatty liver disease
*MDCT*: Multidetector computed tomography
*Mets*: Metabolic syndrome
*MI*: Myocardial infarction
*MMP*: Matrix metalloproteinase
*MPR*: Myocardial perfusion reserve
*MRI*: Magnetic resonance imaging
*NAFLD*: Non-alcoholic fatty liver disease
*NASH*: Non-alcoholic steatohepatitis
*NFS*: NAFLD fibrosis score
*NF-κB*: Nuclear factor-κB
*NSTEMI*: Non-ST-segment elevation myocardial infarction
*OCAD*: Obstructive coronary artery disease
*OCT*: Optical coherence tomography
*OR*: Odds ratio
*PAI-1*: Plasminogen activator inhibitor-1
*PCI*: Percutaneous coronary intervention
*PCSK9-i*: Proprotein convertase subtilisin/Kexin type 9 inhibitor
*PPAR-γ*: Peroxisome proliferator-activated receptor-γ
*PTCA*: Percutaneous transluminal coronary angioplasty
*RCT*: Randomized controlled trial
*sdLDL*: Small dense low-density lipoprotein
*STEMI*: ST-segment elevation myocardial infarction
*T2DM*: Type 2 diabetes mellitus
*TE-CAP*: Transient elastography-Controlled attenuation parameter
*TG*: Triglyceride
*TIA *: Transient ischemic attack
*TRLs*: Triglyceride-rich lipoproteins
*TyG*: Triglyceride-glucose index
*UA*: Unstable angina
*VAT*: Visceral adipose tissue
*VEGF*: Vascular endothelial growth factor
*γ-GTP*: γ-Glutamyl transpeptidase
